# Anesthetic Management of an Urgent Hip Fracture Repair in a Nonagenarian With Critical Aortic Stenosis

**DOI:** 10.7759/cureus.109224

**Published:** 2026-05-19

**Authors:** Benjamin A Koerper, Karl P Koerper

**Affiliations:** 1 Medicine, Burrell College of Osteopathic Medicine, Las Cruces, USA; 2 Anaesthesiology, Three Crosses Regional Hospital, Las Cruces, USA

**Keywords:** anesthetic induction, aortic stenosis (as), critical aortic stenosis, elderly hip fracture, elderly patient care, geriatric anesthesia, high-risk anesthesia, perioperative management, shared decision making, valvular heart disease

## Abstract

Severe aortic stenosis presents substantial anesthetic risk because fixed left ventricular outflow obstruction limits the ability to compensate for perioperative hemodynamic changes. These risks are further amplified in elderly patients with additional valvular disease and limited physiologic reserve who require urgent noncardiac surgery. We report the anesthetic management of a 96-year-old woman with critical aortic stenosis and concomitant mitral valve disease who required urgent hip fracture repair. Preoperative echocardiography demonstrated severe valvular calcification with a markedly reduced aortic valve area and elevated transvalvular gradients consistent with critical aortic stenosis. General anesthesia was induced with etomidate and fentanyl, with invasive arterial monitoring and phenylephrine infusion utilized to maintain coronary perfusion pressure and hemodynamic stability throughout the procedure. Multimodal postoperative pain management and close postoperative monitoring were employed to facilitate recovery while minimizing cardiovascular stress. The patient tolerated the procedure without intraoperative hemodynamic instability and experienced an uncomplicated postoperative recovery. This case illustrates key physiologic principles in the anesthetic management of severe aortic stenosis and highlights the importance of multidisciplinary decision-making and shared informed consent when high-risk patients require urgent surgical intervention.

## Introduction

Aortic stenosis (AS) is one of the most common valvular heart diseases affecting elderly populations, with prevalence increasing significantly with age [1]. Degenerative calcification of the aortic valve leads to progressive obstruction of left ventricular outflow, ultimately resulting in reduced cardiac output and increased myocardial oxygen demand [2]. Severe AS produces a fixed obstruction to ventricular ejection, limiting the heart’s ability to respond to physiologic stress and increasing susceptibility to hypotension, myocardial ischemia, and arrhythmias during anesthesia [2,3].

Historically, severe AS was considered a major contraindication to elective noncardiac surgery due to the elevated risk of perioperative cardiac complications [4]. However, elderly patients with hip fractures often require urgent surgical repair before definitive cardiac intervention can be performed.

Hip fractures represent a common surgical emergency in geriatric populations and are associated with increased morbidity and mortality if operative repair is delayed [5,6]. When severe AS coexists with urgent orthopedic pathology, clinicians must carefully balance the risks of surgery against the complications associated with prolonged immobilization. Careful anesthetic planning and meticulous hemodynamic management are essential in these situations to maintain coronary perfusion and prevent rapid changes in systemic vascular resistance [7,8].

The authors obtained written authorization under the Health Insurance Portability and Accountability Act (HIPAA) from the patient for publication of this case report.

## Case presentation

A 96-year-old woman presented to the emergency department after a fall at home that resulted in a displaced left femoral neck fracture. Prior to the injury, she lived independently and ambulated without assistance, maintaining full autonomy in her daily activities. At the time of presentation, she was alert, oriented, and able to fully participate in medical decision-making.

Her medical history was notable for obstructive sleep apnea, prior right hip surgery, lumbar kyphoplasty, and known valvular heart disease. She denied any history of coronary artery disease, prior cerebrovascular accident, or chronic pulmonary disease. On initial evaluation, the patient appeared frail but was hemodynamically stable. Physical examination revealed a loud systolic murmur consistent with underlying valvular disease, along with localized tenderness of the left hip.

**Figure 1:**
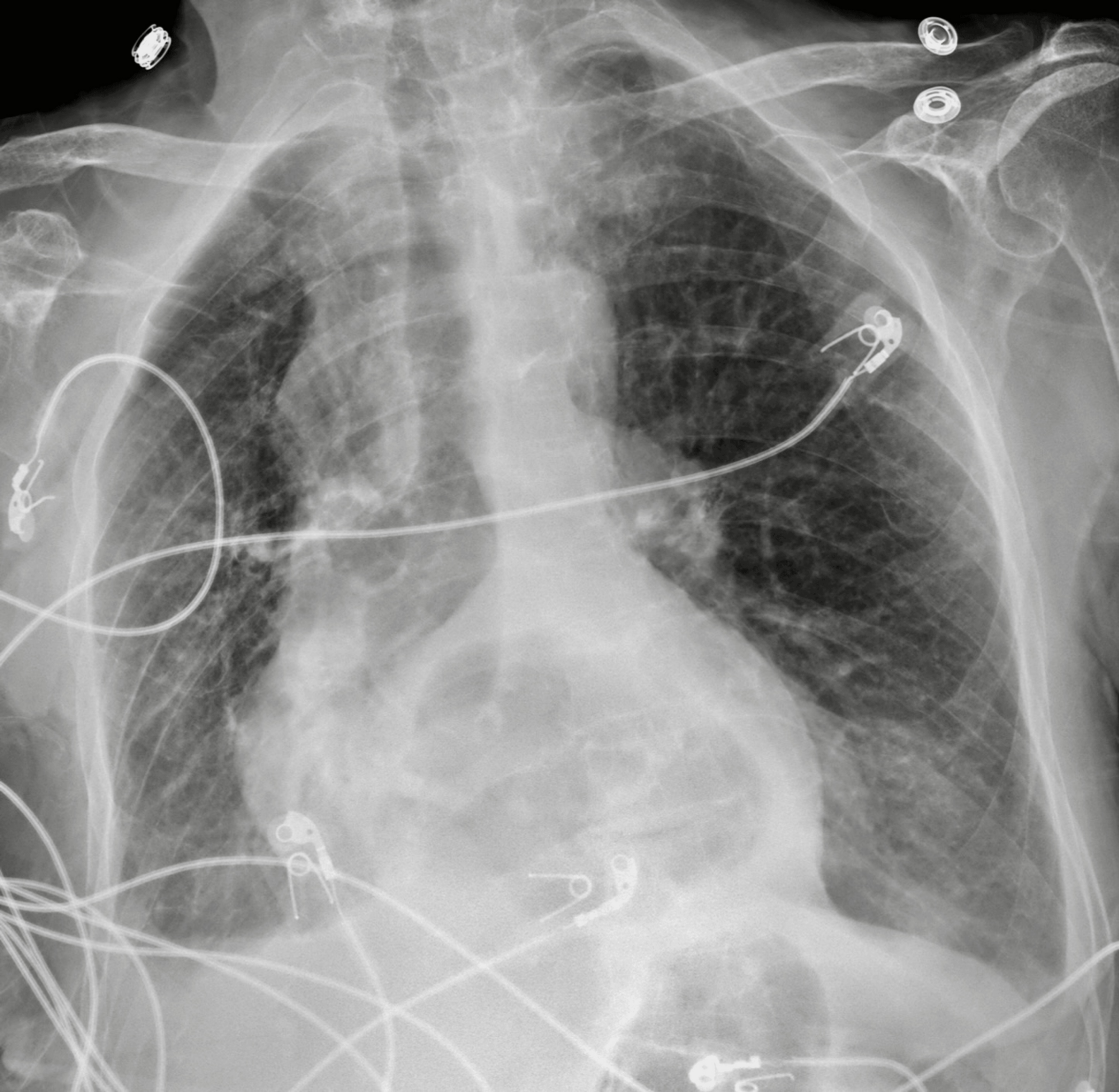
Portable anteroposterior chest radiograph Demonstrates mild interstitial pulmonary edema, right pleural effusion, and enlarged cardiomediastinal silhouette.

Baseline laboratory studies demonstrated hypoalbuminemia and mild electrolyte abnormalities, findings consistent with frailty and limited physiologic reserve. Chest radiography demonstrated mild interstitial pulmonary edema, a right pleural effusion, and an enlarged cardiomediastinal silhouette (Figure 1).

**Figure 2:**
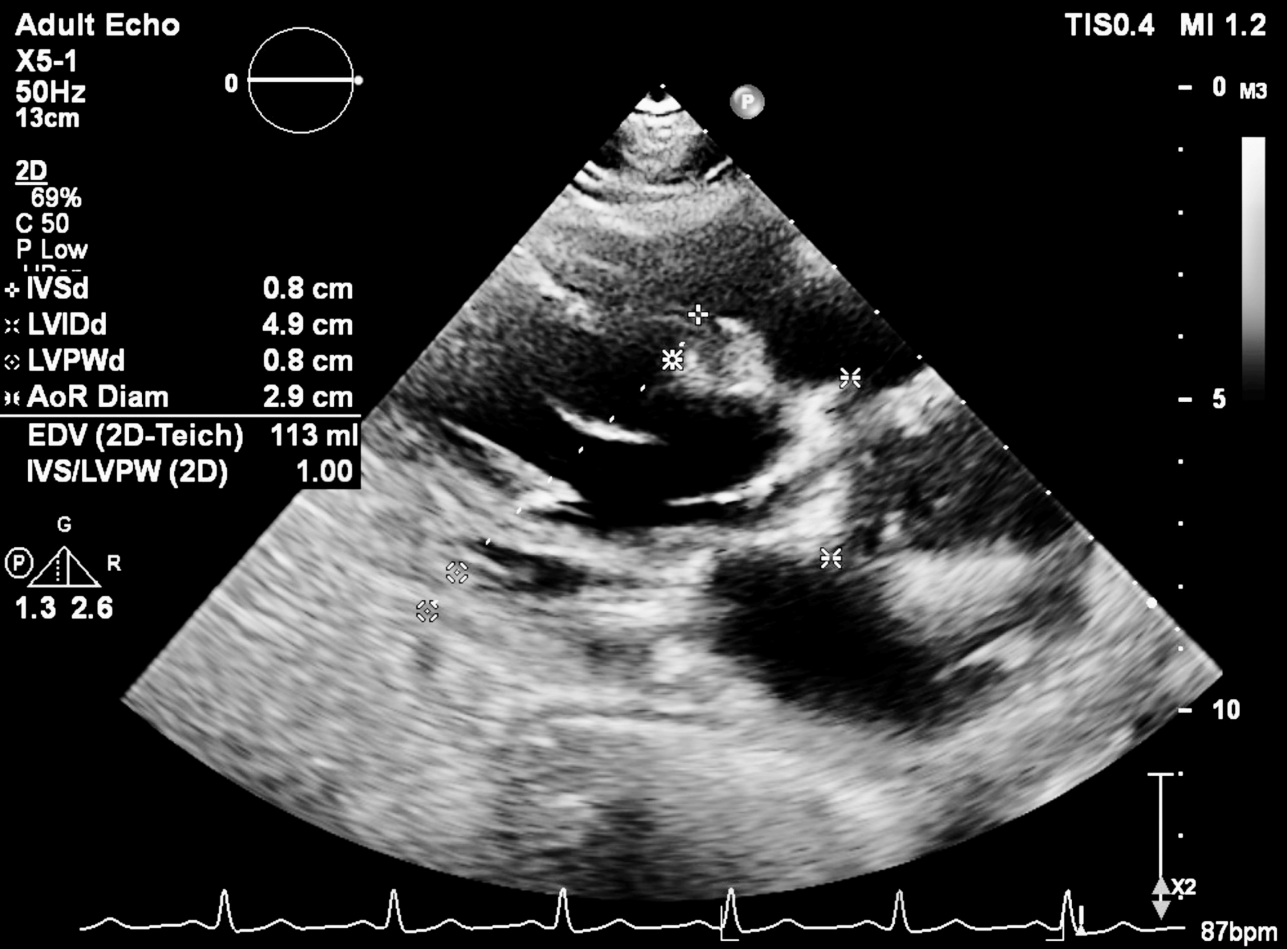
Parasternal long-axis echocardiographic view Demonstrates severe calcification and restricted opening of the aortic valve consistent with critical aortic stenosis.

Transthoracic echocardiography demonstrated critical AS with severe valvular calcification (Figure 2).

**Figure 3:**
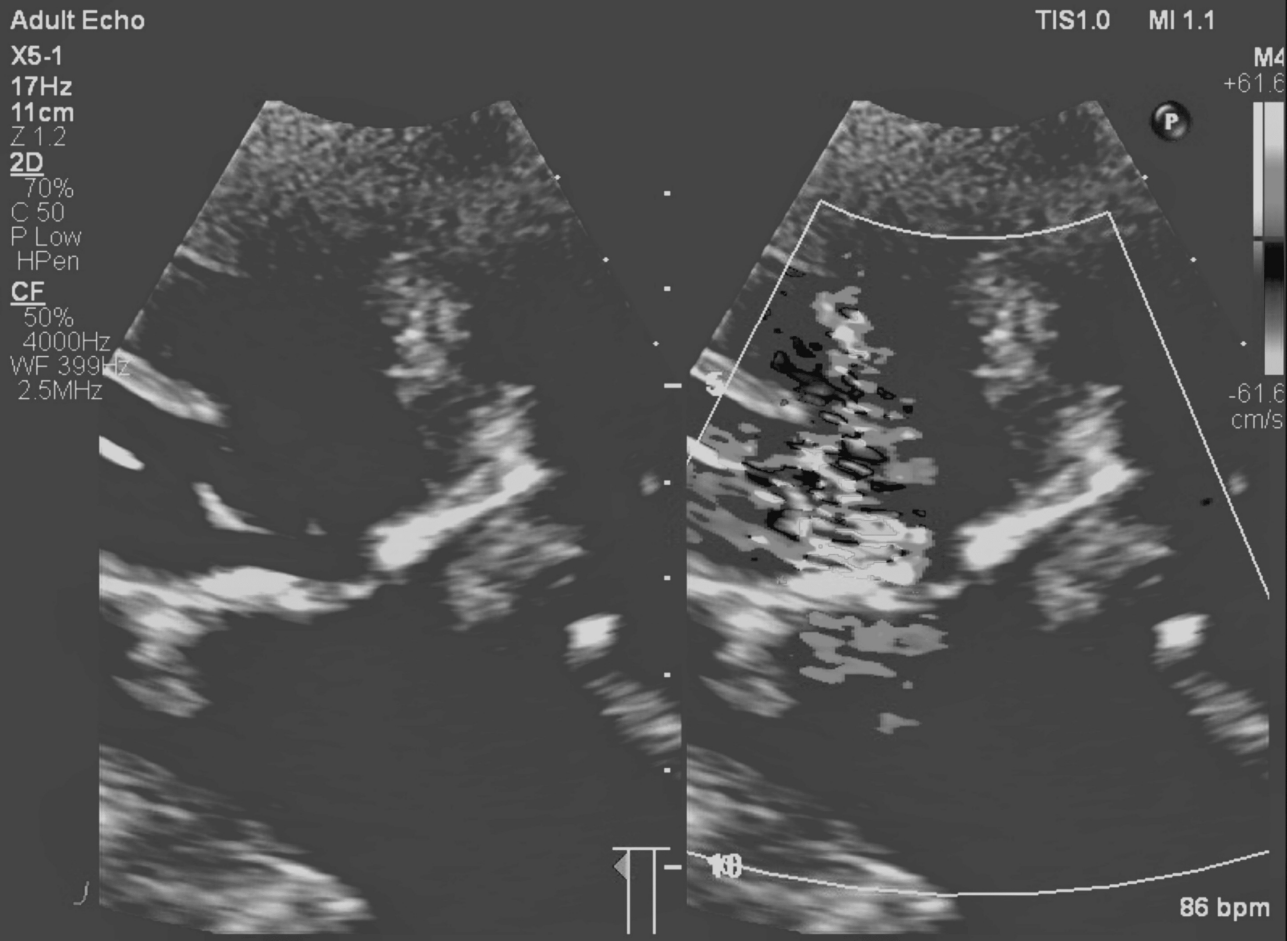
Color Doppler echocardiography demonstrating turbulent systolic flow across the aortic valve

Color Doppler imaging demonstrated turbulent systolic flow across the aortic valve consistent with severe valvular obstruction (Figure 3).

**Figure 4:**
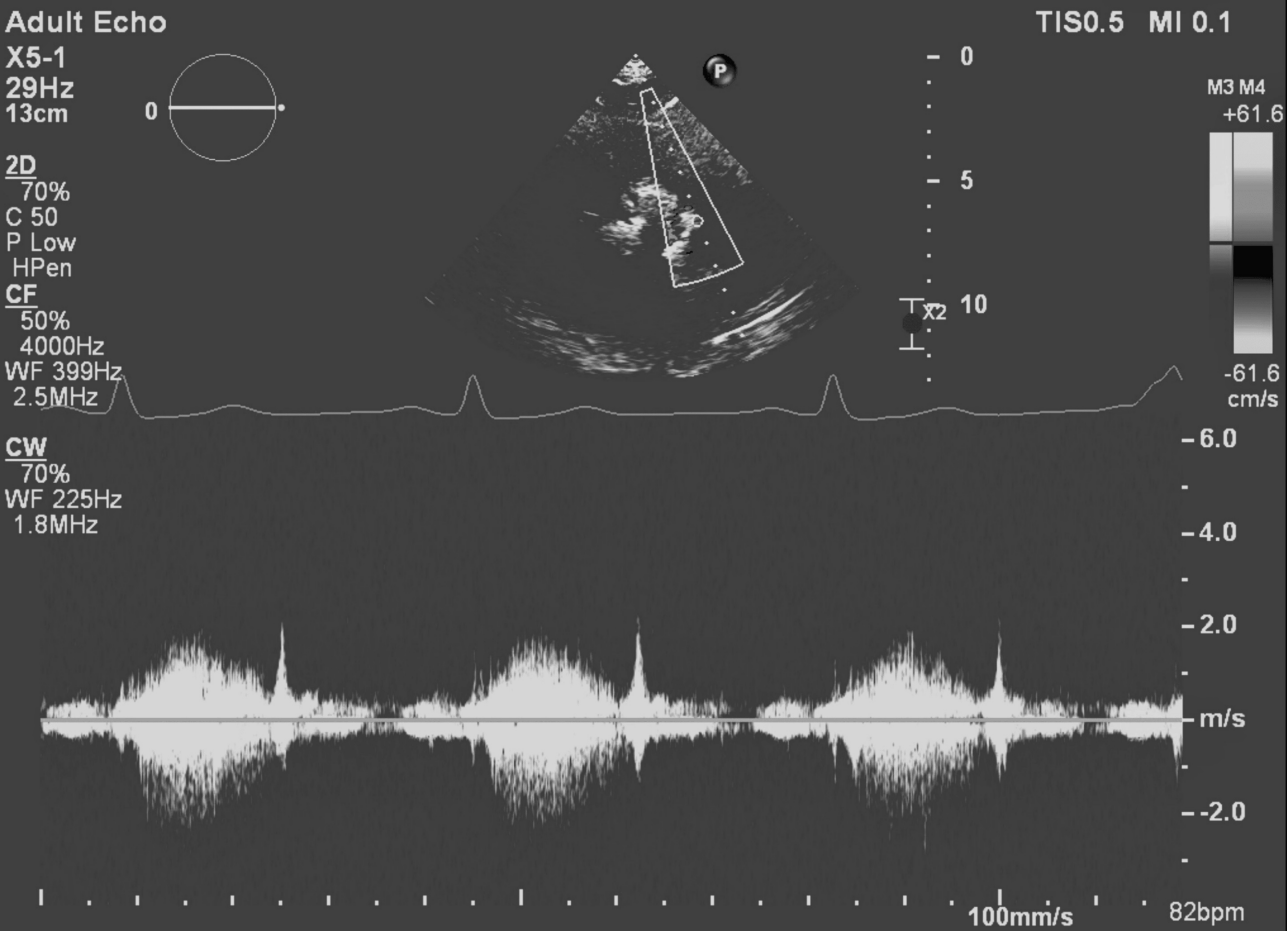
Continuous-wave Doppler interrogation across the aortic valve Demonstrates elevated transvalvular velocities consistent with severe aortic stenosis.

Continuous-wave Doppler interrogation demonstrated markedly elevated transvalvular velocities (Figure 4).

Echocardiographic measurements demonstrated critical AS with an aortic valve area of 0.43 cm², a peak transvalvular gradient of 56 mmHg, a mean gradient of 39 mmHg, and a peak velocity of 3.75 m/s. Left ventricular systolic function was preserved with an estimated ejection fraction of approximately 50%, although diastolic filling was severely constrained by valvular pathology. The left atrium was markedly dilated.

Additional valvular findings included moderate-to-severe mitral regurgitation and moderate mitral stenosis with an estimated mitral valve area of approximately 1.0 cm². Right ventricular systolic function was mildly reduced. Estimated right ventricular systolic pressure was 40-45 mmHg, consistent with moderate pulmonary hypertension.

The patient was evaluated by cardiology and structural heart specialists and determined not to be a candidate for surgical aortic valve replacement or transcatheter aortic valve replacement due to advanced age, severe multivalvular disease, extensive valvular calcification, frailty, and overall procedural risk. Balloon aortic valvuloplasty was considered but deferred because the anticipated procedural risks were felt to outweigh the potential short-term benefit in the setting of extensive calcific valvular disease and advanced age.

Urgent operative fixation was recommended to restore mobility and reduce the complications associated with prolonged immobilization. Multidisciplinary evaluation involving cardiology, anesthesiology, and orthopedic surgery determined that the patient faced a markedly elevated perioperative risk due to critical AS, advanced age, pulmonary hypertension, and concomitant mitral valve disease. However, nonoperative management of the displaced femoral neck fracture would likely have resulted in permanent immobility and with little chance of regaining independent function. Prior to the injury, the patient had been living independently and maintained full autonomy in her daily activities. After detailed counseling regarding the potential risks and benefits of surgery, the patient demonstrated clear decision-making capacity and, together with her family, elected to proceed with operative management in order to preserve functional mobility and maintain independence.

Anesthetic management

Given the patient’s critical AS, pulmonary congestion, and additional valvular disease, anesthetic planning focused on maintaining coronary perfusion pressure, avoiding hypotension and tachycardia, and minimizing abrupt changes in preload and afterload [1-3]. Prior to induction, invasive brachial arterial blood pressure monitoring was established, and central venous access via the right internal jugular vein was obtained to facilitate administration of vasoactive medications. Standard American Society of Anesthesiologists (ASA) monitors, bispectral index monitoring, and quantitative neuromuscular monitoring were applied.

General anesthesia was induced using etomidate and fentanyl in order to minimize myocardial depression and systemic vasodilation. Neuromuscular blockade was achieved with rocuronium, and endotracheal intubation was performed using video laryngoscopy. Hemodynamic goals included maintaining sinus rhythm, avoiding tachycardia, preserving preload, and maintaining mean arterial pressure sufficient to support coronary perfusion. A phenylephrine infusion was initiated early during induction and titrated throughout the procedure to minimize reductions in systemic vascular resistance and maintain hemodynamic stability.

Anesthesia was maintained with a low concentration of volatile anesthetic with careful attention to hemodynamic stability. Continuous invasive arterial monitoring allowed rapid detection and treatment of blood pressure changes throughout the procedure. Sinus rhythm was preserved throughout the case, and significant hypotension or tachycardia was avoided. Fluid administration was judicious given the patient’s preload dependence and limited tolerance for volume overload, with approximately 1 liter of crystalloid administered intraoperatively. Tranexamic acid was administered to minimize intraoperative blood loss. Total operative duration and intraoperative blood loss were limited, and urine output remained adequate throughout the procedure.

The surgical procedure consisted of bipolar hemiarthroplasty of the left hip and proceeded without significant hemodynamic instability. Estimated blood loss was minimal and no transfusion was required. Neuromuscular blockade was reversed at the conclusion of the procedure, and the patient was successfully extubated in the operating room before transfer to the post-anesthesia care unit with close monitoring and intensive care availability.

## Discussion

This case illustrates the complex clinical, anesthetic, and ethical considerations that arise when high-risk patients with critical AS require urgent noncardiac surgery. While the physiologic principles underlying anesthetic management of severe AS are well established, this case is notable because of the patient’s advanced age, preserved preinjury functional independence, multivalvular pathology, and the multidisciplinary decision to proceed with urgent operative repair despite substantial perioperative cardiovascular risk. Severe AS presents a significant anesthetic challenge because the fixed obstruction to left ventricular outflow limits the heart’s ability to increase cardiac output in response to physiologic stress. As a result, patients are particularly vulnerable to hypotension, tachycardia, and myocardial ischemia during anesthesia and surgery [1,3]. Careful perioperative planning is therefore essential to maintain coronary perfusion pressure and avoid abrupt changes in preload, afterload, or heart rate.

In patients with severe AS, anesthetic management must account for the fixed obstruction to left ventricular outflow and the resulting dependence on adequate preload, sinus rhythm, and maintenance of systemic vascular resistance to preserve coronary perfusion pressure. Hypotension and tachycardia can significantly compromise myocardial oxygen supply-demand balance in this population and may precipitate hemodynamic collapse during anesthesia [1,9]. The coexistence of moderate mitral stenosis, moderate-to-severe mitral regurgitation, pulmonary hypertension, and advanced age further increased physiologic complexity and limited cardiovascular reserve in this patient. For this reason, invasive arterial monitoring, careful titration of anesthetic agents, and early use of vasopressors are commonly recommended strategies to maintain hemodynamic stability during induction and throughout the procedure.

Urgent hip fracture repair presents an additional clinical dilemma in elderly patients with severe AS. Hip fractures are associated with substantial morbidity and mortality, particularly when operative repair is delayed. Nonoperative management often results in prolonged immobility, increased risk of thromboembolism, pneumonia, pressure injuries, and loss of functional independence. Previous studies have demonstrated that timely surgical repair improves outcomes and may reduce overall mortality in elderly patients with hip fractures [4]. Consequently, clinicians must carefully balance the elevated perioperative cardiac risk against the significant complications associated with prolonged immobilization.

In the present case, multidisciplinary evaluation determined that the patient faced a markedly elevated perioperative risk due to advanced age, pulmonary hypertension, severe AS, and concomitant mitral valve disease. Despite these risks, the anticipated consequences of nonoperative management were substantial, particularly given the patient’s previously independent functional status. Contemporary valvular heart disease guidelines emphasize individualized risk assessment and shared decision-making when managing patients with severe AS who require noncardiac surgery [7,9]. These discussions should incorporate not only physiologic risk but also the patient’s baseline functional status, quality-of-life expectations, and personal goals of care.

Anesthetic management in this case focused on preserving hemodynamic stability throughout induction, maintenance, and emergence. Etomidate was selected because of its relative hemodynamic stability, while phenylephrine infusion was used to maintain coronary perfusion pressure and systemic vascular resistance. Continuous invasive arterial monitoring allowed rapid identification and treatment of hemodynamic fluctuations throughout the procedure. Careful fluid administration was necessary because excessive volume administration risked worsening pulmonary congestion, while inadequate preload could precipitate cardiovascular collapse in the setting of critical AS and mitral stenosis.

Respecting patient autonomy played a central role in the decision to proceed with surgery in this case. The patient demonstrated full decision-making capacity and was counseled extensively regarding the potential risks associated with anesthesia and surgical intervention. After discussion with both the care team and family members, the patient expressed a clear preference to pursue operative management in hopes of preserving mobility and maintaining independence. In this context, proceeding with surgery represented a patient-centered approach that aligned medical decision-making with the patient’s expressed values and goals.

Postoperative management focused on continued hemodynamic monitoring, multimodal pain control, pulmonary hygiene, early mobilization, and prevention of delirium and deconditioning. The patient remained hemodynamically stable postoperatively and did not experience major cardiovascular complications. Careful postoperative monitoring was particularly important given the patient’s critical valvular disease, pulmonary hypertension, advanced age, and limited physiologic reserve.

The patient recovered without major perioperative cardiovascular complications and experienced an uncomplicated postoperative course. At the five-month follow-up, she had celebrated her 97th birthday, was ambulating with a walker, and was living with family while continuing rehabilitation. This favorable outcome highlights that carefully selected high-risk patients with severe AS may still derive meaningful functional benefit from operative management when perioperative care is meticulously planned.

Overall, this case highlights the importance of individualized clinical decision-making in high-risk surgical patients. Although severe AS substantially increases perioperative risk, this risk must be weighed against the functional consequences of nonoperative management, particularly in elderly patients with previously independent lifestyles. When multidisciplinary planning, careful anesthetic management, and shared decision-making are employed, urgent surgical intervention may still provide meaningful improvements in mobility, independence, and quality of life even in patients with significant cardiovascular comorbidities [4,7,9].

## Conclusions

This case highlights the importance of individualized decision-making when managing elderly patients with significant comorbidities who require urgent surgical intervention. Although critical AS confers substantial perioperative risk, treatment decisions must also consider the patient’s baseline functional status, goals of care, and quality of life. In this case, careful multidisciplinary evaluation and thorough informed consent allowed the patient to participate fully in the decision-making process. Respecting the patient’s autonomy and her desire to preserve independence ultimately guided the decision to proceed with surgery despite the elevated risk. The procedure was completed without complications, and the patient recovered well and was successfully discharged from the hospital. This case underscores the importance of aligning clinical management with patient-centered values while balancing physiologic risk and functional outcomes.
